# Ribosomal protein L22 like 1: a promising biomarker for lung adenocarcinoma

**DOI:** 10.7150/jca.91759

**Published:** 2024-03-11

**Authors:** Yahua Wu, Na Yao, Bin Du, Yingjiao Zhu, Xiaohui Ji, Chengliu Lv, Jinhuo Lai

**Affiliations:** 1 Department of Medical Oncology, Fujian Medical University Union Hospital, No. 29 Xinquan Street, Fuzhou, 350000, Fujian, China.; 2 Department of Medical Oncology, Chongqing University Cancer Hospital, Chongqing, 400030, China.

**Keywords:** RPL22L1, Biomarker, Lung adenocarcinoma, Prognosis, TME

## Abstract

No studies have reported the effect of ribosomal protein L22 like 1 (RPL22L1) in lung adenocarcinoma (LUAD). Therefore, we aimed to systematically investigate the role of RPL22L1 in LUAD. The expression of RPL22L1 was analyzed using TCGA, GEO, TIMER, UALCAN databases, and validated by immunohistochemistry (IHC). Gene methylation analysis was performed using the UALCAN, GSCA and MethSurv databases. The immune infiltrates were investigated using the Single Sample Gene Set Enrichment Analysis (ssGSEA), TIMER database, and TISCH database. The results demonstrated that RPL22L1 was up-regulated in LUAD, and verified by IHC. Kaplan-Meier analysis suggested that patients with high RPL22L1 expression had poor prognosis. Multivariate analysis confirmed that RPL22L1 was an independent prognostic factor. Furthermore, RPL22L1 overexpression was associated with hypomethylation, and two CpGs of RPL22L1 were significantly associated with prognosis. Up-regulated RPL22L1 was enriched in MYC targets, E2F targets, G2M checkpoint, mTORC1 signaling, cell cycle, and so on. Moreover, RPL22L1 expression was negatively correlated with immune cell infiltration, and patients with high RPL22L1 expression had lower immune, stromal, and estimate scores. Single-cell analysis suggested that RPL22L1 might have a potential function in the tumor microenvironment (TME) of LUAD. In conclusion, RPL22L1 may be a promising biomarker for LUAD.

## Introduction

Lung cancer is one of the most prevalent cancers in the world.^1^ Lung adenocarcinoma (LUAD) is a common pathologic subtype of lung cancer.^2^ Despite targeted therapy and immunotherapy improving the survival of LUAD patients, the prognosis is still unsatisfactory.^3^, ^4^ Therefore, it remains urgent to explore more molecular targets.

Ribosomal proteins (RPs) are components of ribosomes and play important regulatory role in ribosome biosynthesis and protein translation. More and more studies have revealed that ribosomal proteins are not only involved in protein synthesis,^5^ but also in the regulation of cell proliferation, migration invasion, apoptosis, cell cycle, and angiogenesis.^6^-^8^ RPL22L1 is a member of the ribosomal protein family and a homologous analog of RPL22.^9^ A increasing number of studies have demonstrated that RPL22L1 promotes to the progression of carcinoma. Chen et al. found that RPL22L1 promoted glioblastoma resistance to temozolomide by activating STAT3 pathway.^10^ Zhang et al. demonstrated that RPL22L1 contributed to sorafenib resistance in hepatocellular carcinoma through the ERK signaling pathway.^11^ In addition, a study by Rao indicated that RPL22L1 was associated with poor prognosis and 5-FU resistance in colorectal cancer.^12^ RPL22L1 was aberrantly expressed in ovarian cancer^13^ and prostate cancer.^14^, ^15^ However, no studies have reported the effect of RPL22L1 in LUAD.

Tumor microenvironment (TME) is a complex and dynamic system mainly containing tumor cells, immune cells, extracellular matrix,^16^ which are strongly related to the development and progression of carcinoma.^17^ Immune cells, such as macrophages, dendritic cells (DCs), natural killer (NK) cells, CD8+ T cells, CD4+ T cells and B cells perform critical roles in tumor growth and metastasis.^18^ It has been found that RPs are involved in regulating immune cell infiltration in the TME.^19^, ^20^ Futhermore, a study has demonstrated that RPL22L1 affects the development of lymphocytes.^21^ Therefore, it is worth investigating whether RPL22L1 is involved in the regulation of the TME, which in turn influences LUAD progression.

Currently, there are fewer studies reported on RPL22L1. The role of RPL22L1 in LUAD and its effect on the TME has not been reported. Our study attempted to systematically reveal the role of RPL22L1 in LUAD and to preliminarily explore its impact on the TME, providing a new biomarker for the diagnosis, treatment, and prognosis of LUAD.

## Material and Methods

### Data acquisition

The mRNA data and the clinical information of LUAD were downloaded from The Cancer Genome Atlas (TCGA) database (https://portal.gdc.cancer.gov/). GSE31210, GSE43458 and GSE115002 were downloaded from the Gene Expression Omnibus (GEO) database (https://www.ncbi.nlm.nih.gov/geo/). Tumor Immune Estimation Resource (TIMER) 2.0 (http://timer.cistrome.org/) was used to analyze the mRNA expression of RPL22L1 in pan-cancer.^22^ The UCALAN (http://ualcan.path.uab.edu/) was utilized to compare protein expression analysis of RPL22L1 in LUAD using The Clinical Proteomic Tumor Analysis Consortium (CPTAC) database.^23^

### Gene mutation and methylation analysis

The Sangerbox 3.0 online tool (http://www.sangerbox.com) was used to analyze the relationship between the genetic mutational landscape and RPL22L1 mRNA expression. RPL22L1 mutation analysis was performed on the cBioPortal online (http://www.cbioportal.org).^24^ The UALCAN database (http://ualcan.path.uab.edu/) was used to explore RPL22L1 promoter DNA methylation levels between LUAD and normal tissues.^25^ The GSCA database was used to evaluate the relationship between RPL22L1 mRNA expression and DNA methylation levels (http://bioinfo.life.hust.edu.cn/GSCA).^26^ The MethSurv database was used to analysis the methylation map of RPL22L1 in LUAD.^27^ We also utilized the MethSurv database to explore the effect of DNA methylation of each CpG in RPL22L1 on the survival of LUAD patients.

### Differentially expressed genes (DEGs) analysis

Based on the median value of mRNA expression of RPL22L1 in TCGA-LUAD, patients were divided into low and high expression groups. We utilized the "DEseq2" package for DEGs.^28^ The criteria of DEGs were absolute log2 fold change (FC) > 1 and adjusted P < 0.05.

### Functional analysis

We downloaded “h.all.v7.2.symbols.gmt” and “c2.cp.kegg.v7.2.symbols.gmt” gene sets from the Molecular Signatures Database (MSigDB) (http://www.gsea-msigdb.org/gsea/index.jsp).^29^ The “clusterProfiler” was used to perform Gene Set Enrichment (GSEA) analysis,^30^ and significantly enriched terms were defined as those having a false discovery rate (FDR) < 0.25 and an adjusted P value < 0.05. Gene Ontology (GO) enrichment and Kyoto Encyclopedia of Genes and Genomes (KEGG) pathway analysis were performed utilizing R package “clusterProfiler” based on DEGs and visualized by R package “ggplot2”.

### Immune cell infiltration analysis

We evaluated the calculation of the stromal score, immune score, and estimated score for each TCGA-LUAD sample by “ESTIMATE” R package. TIMER was utilized to analyze the correlation between RPL22L1 in LUAD and immune cells (B cells, CD4+ T cells, CD8+ T cells, neutrophils, macrophages, and dendritic cells) and tumor purity. The single sample Gene Set Enrichment Analysis (ssGSEA) was used to identify the composition of 24 infiltrating immune cells.^31^

### Single cells analysis

The Tumor Immune Single Cell Center (TISCH) database is a single‐cell RNA database (http://tisch.comp-genomics.org) that is used to assess the expression level of RPL22L1 of different cell types in the tumor microenvironment. 32

### Drug sensitivity

The half-maximal inhibitory concentrations (IC50) of chemotherapeutic or targeted drugs were calculated using the pRRophetic R package.^33^ Patients were divided into RPL22L1 high and low expression groups according to median expression values, and differences in the IC50 of these drugs between the two groups were analyzed using the Wilcoxon test.

### Immunohistochemistry (IHC)

A tissue microarray containing 90 pairs of LUAD and adjacent normal tissues was purchased from Shanghai Outdo Biotech Company. RPL22L1 antibody for IHC staining was obtained from Abcam (ab234792). The IHC scores were independently assessed by two experienced pathologists. Staining intensity score was defined as 0 = negative staining, 1 = weak staining, 2 = moderate staining, and 3 = strong staining. The positive cells score: 0: < 1%, 1: 1-25%, 2: 25-50%, 3: 50-75% and 4: > 75%. The IHC score was calculated as positive cells score × staining intensity score.

### Statistical analysis

Statistical analysis was carried by the R software (version 4.2.2) and SPSS (version 25.0). The Wilcoxon rank‐sum test was used for continuous data and Chi-square test or Fisher's exact test was used for categorical variables. The survival curve was plotted using the Kaplan-Meier method with the log-rank test. Univariate and multivariate Cox regression analyses were used to determine independent prognostic factors. The diagnostic value of PRL22L1 was verified by the receiver operating characteristic (ROC) curve. All analyses were two‐sided, and statistical significance was P value less than 0.05.

## Results

### RPL22L1 was up-regulated in LUAD and pan-cancer

**Figure 1:**
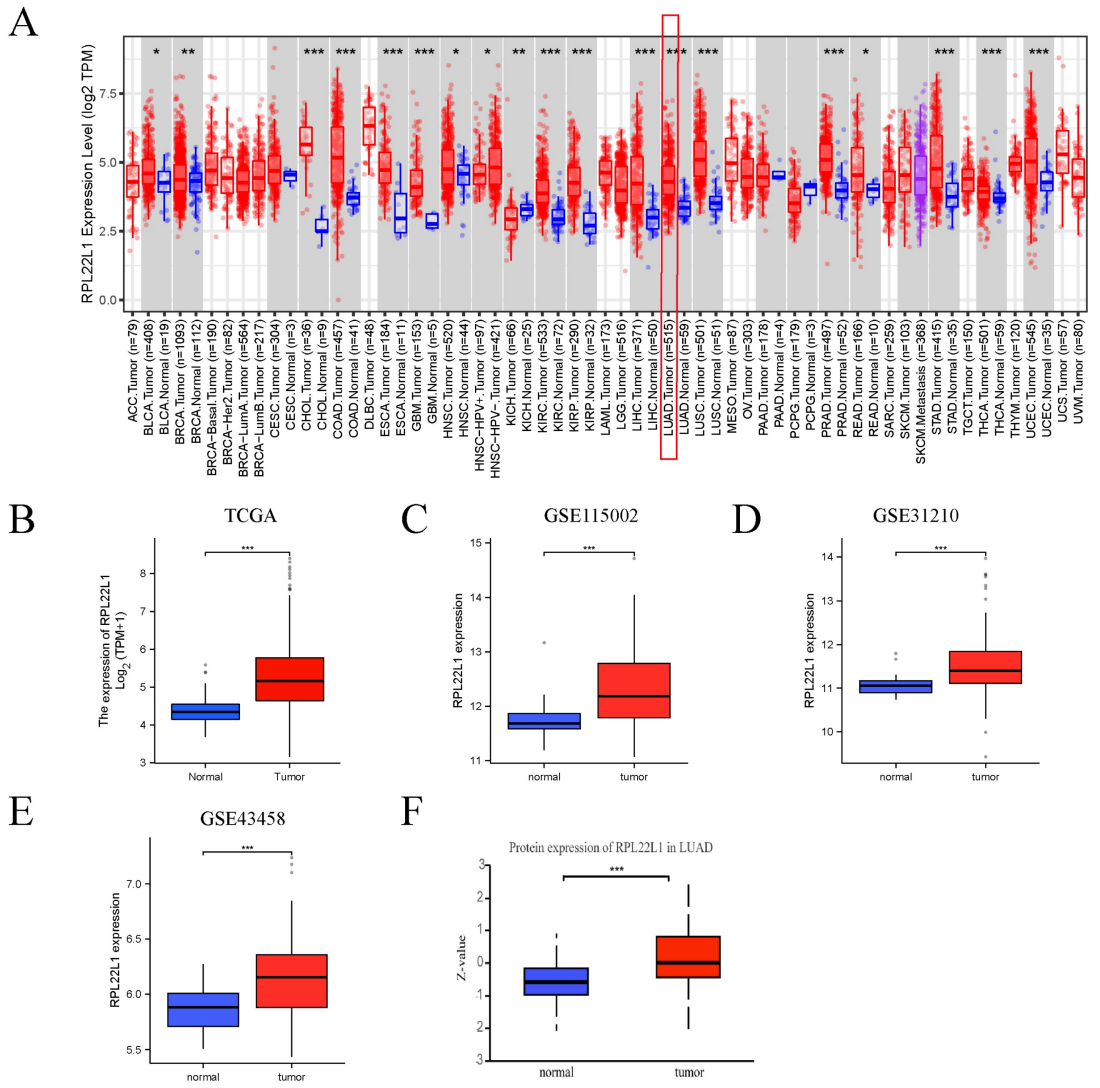
The expression of RPL22L1 in LUAD and pan-carcinoma. Difference in the level of RPL22L1 mRNA expression between LUAD and normal tissues in pan-cancer using the TIMER database (A). Difference in expression of RPL22L1 between LUAD and normal tissues in TCGA (B), GSE115002 (C), GSE31210 (D), GSE43458 (E) datasets. The protein expression level of RPL22L1 between LUAD and normal tissues using CPTAC database (F). *P < 0.05; **P < 0.01; ***P < 0.001; ****P < 0.0001.

Firstly, pan-cancer analysis showed that mRNA levels of RPL22L1 were up-regulated in most tumors, including LUAD. Next, we estimated the mRNA level of RPL22L1 in the TCGA database, and the result showed that the mRNA of RPL22L1 was highly expressed in LUAD. To further confirm the expression level of RPL22L1, we included three other independent datasets (GSE31210, GSE43458 and GSE115002), which also confirmed that the mRNA of RPL22L1 was highly expressed in LUAD. Based on CPTAC database, the protein expression level of RPL22L1 was elevated in LUAD compared to normal tissues (Figure 1).

### Relationship between RPL22L1 and clinicopathologic variables

**Figure 2:**
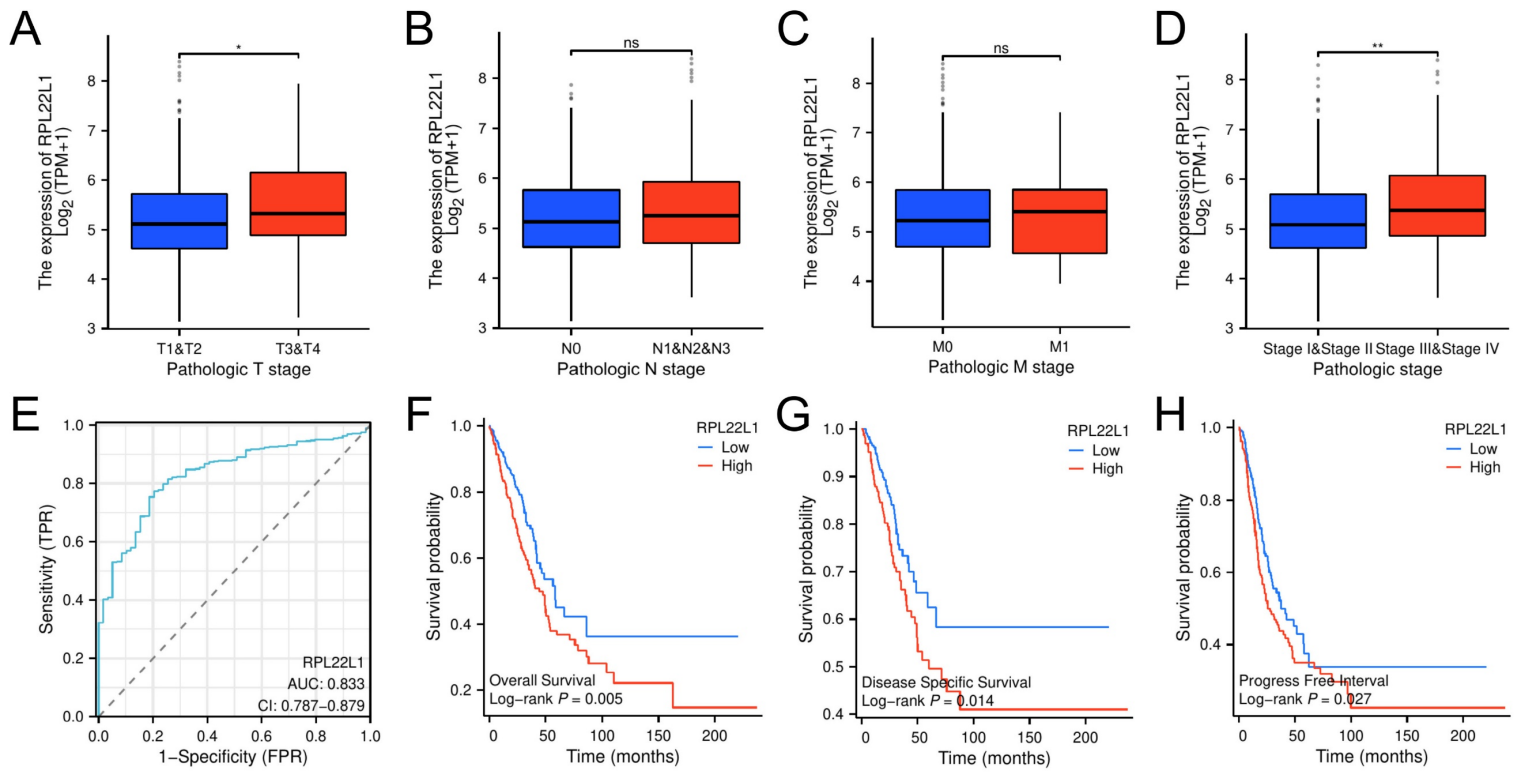
Relationship between RPL22L1 and clinicopathologic variables in LUAD. Difference in the level of RPL22L1 mRNA expression in T stage (A), N stage (B), M stage (C), pathologic stage (D). The receiver operating characteristic (ROC) curve for RPL22L1 (E). The overall survival (F), disease-specific survival (G) and progression-free interval (H) between high RPL22L1 and low RPL22L1 expression groups. *P < 0.05; **P < 0.01.

The median value of RPL22L1 expression level was used as a threshold to categorize LUAD patients into RPL22L1 high expression group (n=270) and low expression group (n=269) in the TCGA database. As shown in Table 1, T3/T4 stage, lymph node metastasis, distant metastasis, and stage III/IV patients accounted for 14.8%, 35.2%, 6.3%, and 24.8%, respectively, in the RPL22L1 high-expression group, whereas it accounted for 10.4%, 29%, 3%, and 16%, respectively, in the low-expression group. Furthermore, the RPL22L1 expression was significantly up-regulated in patients with T3-4 and III-IV stage compared to patients with T1-2 and I-II stage (Figure 2A-D). In addition, we used the ROC curve to verify the diagnostic value of RPL22L1 for LUAD. The result indicated that the area under the ROC curve of RPL22L1 was 0.833 (95% CI: 0.787-0.879) (Figure 2E).

### High expression of RPL22L1 correlated with poor prognosis

We assessed the prognostic value of RPL22L1 in LUAD in the TCGA database. Patients with high RPL22L1 expression had worse overall survival (Figure 2F), disease-specific survival (Figure 2G), and progression-free interval (Figure 2H) than patients with low RPL22L1 expression. In addition, univariate analysis showed that RPL22L1 expression, T stage, N stage, M stage and pathologic stage were associated with OS. Multivariate analysis indicated that RPL22L1 expression was an independent prognostic factor for LUAD (Table 2).

### IHC experimental verification

**Figure 3:**
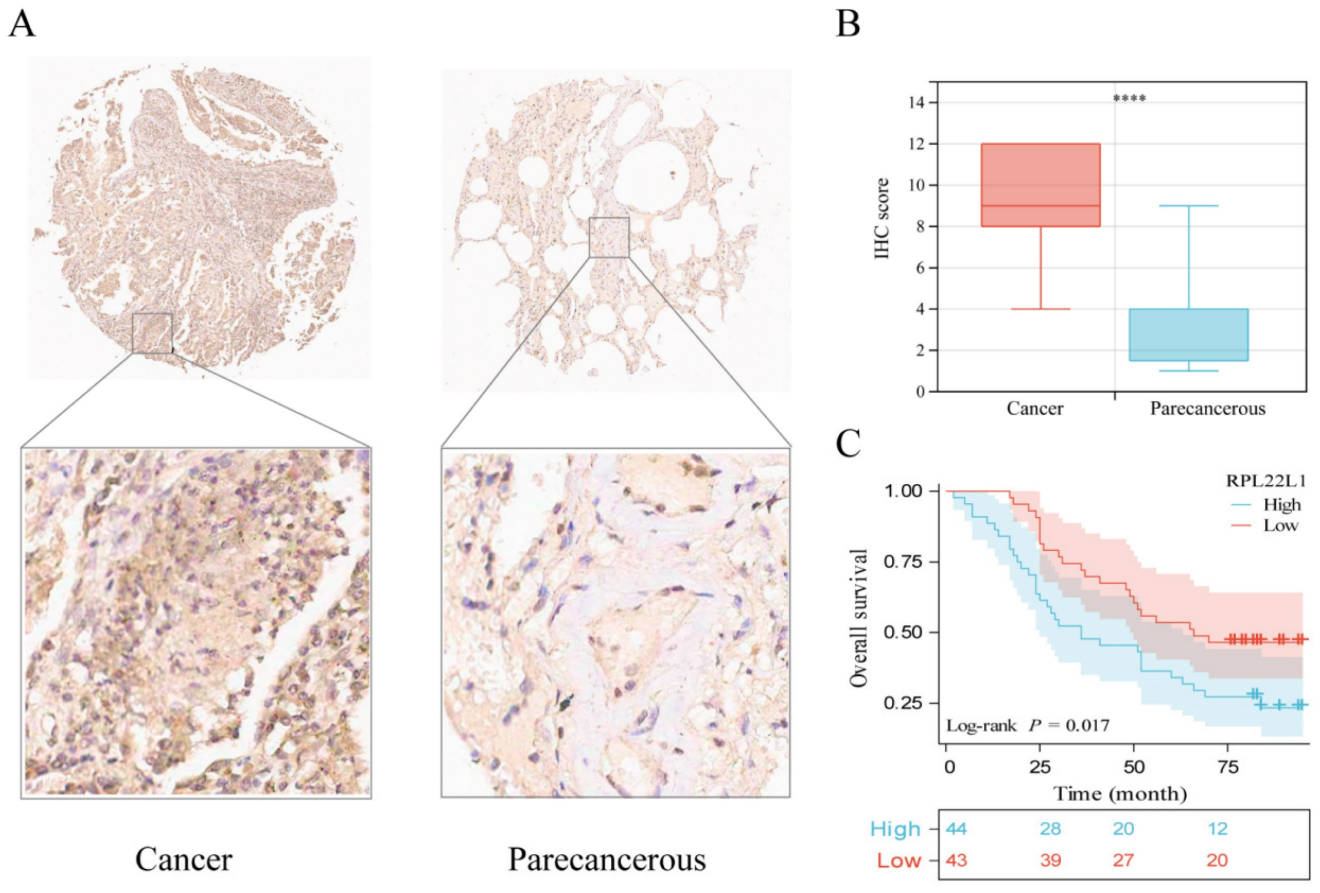
RPL22L1 protein expression in LUAD and normal tissues by IHC. The representative images of RPL22L1 staining in LUAD and adjacent tissues (A). IHC score between LUAD and adjacent tissues (B). The OS curve of LUAD patients according to RPL22L1 IHC score (C).

LUAD tissue microarray was used for IHC to assess RPL22L1 protein levels (87 pairs of carcinomas/paracarcinomas were included in further analyses after exclusion of missing spots). Representative IHC images of RPL22L1 in LUAD and paracancerous tissues are shown in Figure 3A. IHC scores were significantly higher in LUAD than in paracancerous tissues (P < 0.0001, Figure 3B). Survival analysis demonstrated that patients with high RPL22L1 expression had worse OS (P = 0.017, Figure 3C). Multivariate analysis also verified that RPL22L1 expression was an independent prognostic factor for LUAD (Table 3).

### Variation and methylation analyses of RPL22L1

**Figure 4:**
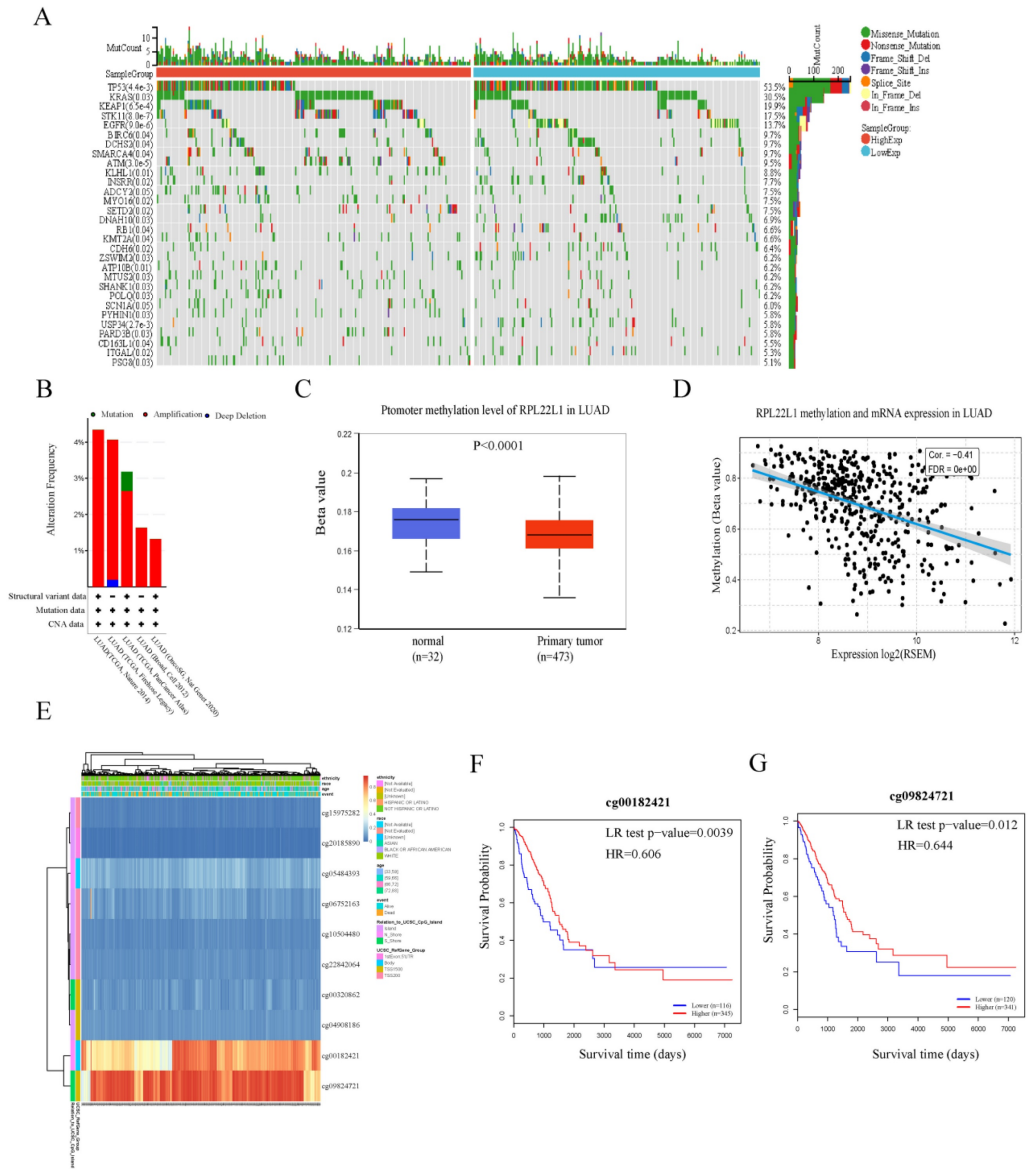
Variation and methylation analyses of RPL22L1 in LUAD. The waterfall plot for the distribution of the top 30 mutated genes with significant differences between the RPL22L1 high and low expression groups (A). The variation of RPL22L1 from the cBioPortal database (B). The DNA methylation levels of RPL22L1 between LUAD and normal tissues using the UALCAN database (C). The relationship between RPL22L1 mRNA expression and DNA methylation (D). The methylation map of RPL22L1 from the MethSurv database (E). Prognostic value of island-cg00182421 (F). Prognostic value of island-cg09824721 (G).

First, we explored the distribution of mutations associated with RPL22L1 expression in the LUAD cohort from the TCGA database. The waterfall plot showed the distribution of the top thirty mutated genes with significant differences between the RPL22L1 high and low expression groups. The top five mutated genes with significant differences between the two groups included TP53, KRAS, KEAP1, STK11 and EGFR (Figure 4A). Moreover, the results from the cBioPortal database showed that the frequency of genetic variation of RPL22L1 was 1.32-4.35% in LUAD, and amplification was the most frequent variation (Figure 4B). Next, we examined the DNA methylation levels of RPL22L1 in LUAD using the UALCAN database, and found that the methylation level of RPL22L1 in LUAD was lower than that in normal tissues (Figure 4C). The GSCA database indicated that RPL22L1 mRNA expression was negatively correlated with DNA methylation levels in LUAD patients (r = -0.41, p < 0.001, Figure 4D). We also obtained the methylation map of RPL22L1 from the MethSurv database and observed that RPL22L1 has ten CpG sites, of which two CpG sites (cg00182421 and cg09824721) were significantly associated with prognosis (Figure 4E-G).

### Functional analysis of RPL22L1

**Figure 5:**
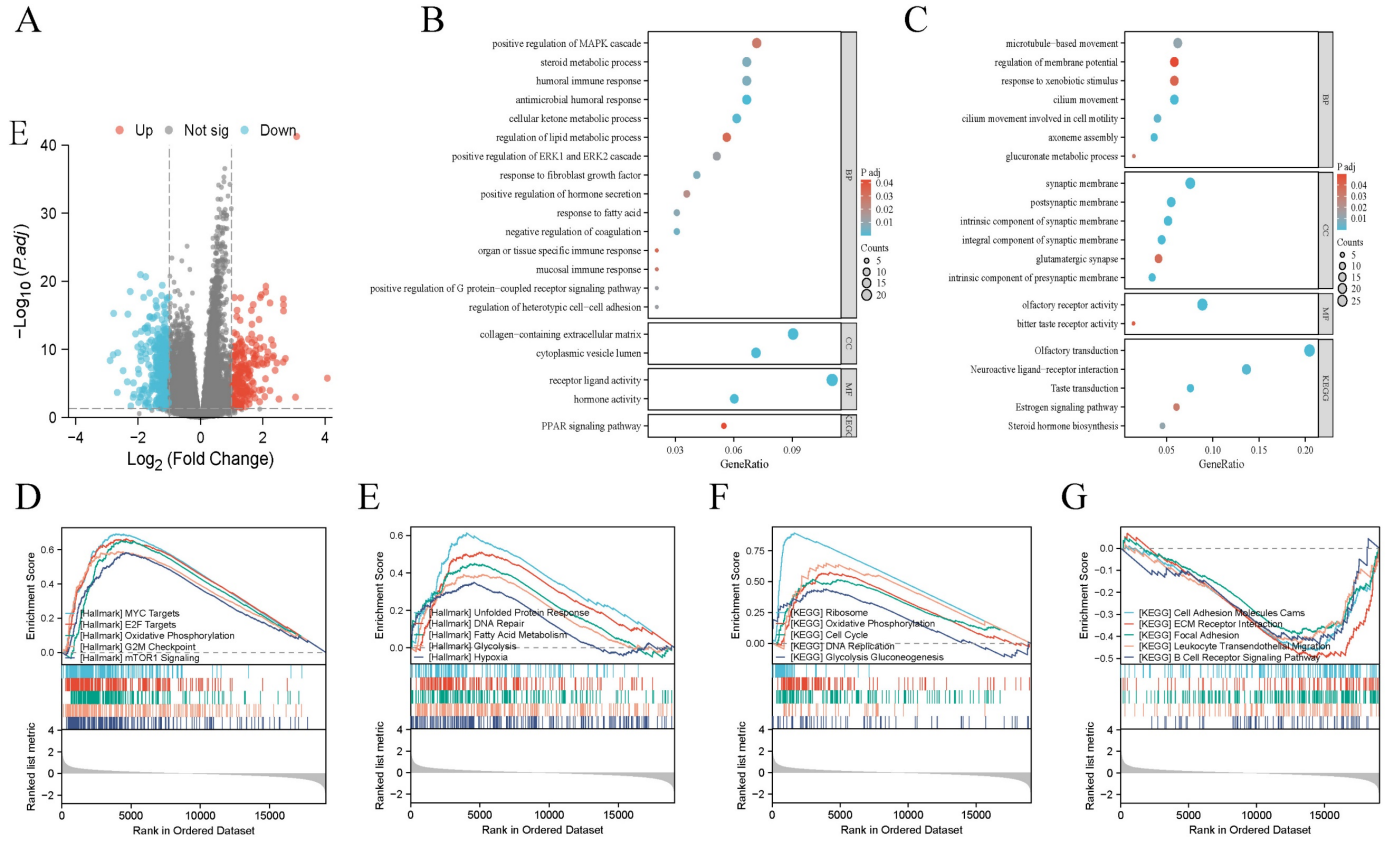
Functional analysis of RPL22L1 in LUAD. Volcano map shows Differentially expressed genes (DEGs) (A). Gene Ontology (GO) enrichment and Kyoto Encyclopedia of Genes and Genomes (KEGG) pathway analysis based on DEGs (B-C). Gene Set Enrichment Analysis (GSEA) (D-G).

We identified 559 DEGs based on the expression of RPL22L1. There were 341 down-regulated genes and 219 up-regulated genes among these DEGs (Figure 5A). GO and KEGG enrichment analyses indicated that up-regulated DEGs were enriched in humoral immune response, positive regulation of MAPK cascade, ERK1 and ERK2 cascade, steroid metabolic process, regulation of lipid metabolic process (Figure 5B), while down-regulated DEGs were enriched in glucuronate metabolic process, microtubule-based movement, steroid hormone biosynthesis, estrogen signaling pathway (Figure 5C). We further conducted GSEA analysis and the results indicated that RPL22L1 was associated with MYC targets, E2F targets, G2M checkpoint, mTORC1 signaling, DNA repair, cell cycle, ECM receptor interaction and B cell receptor signaling pathway (Figure 5D-G).

### Immune infiltration analysis of RPL22L2

**Figure 6:**
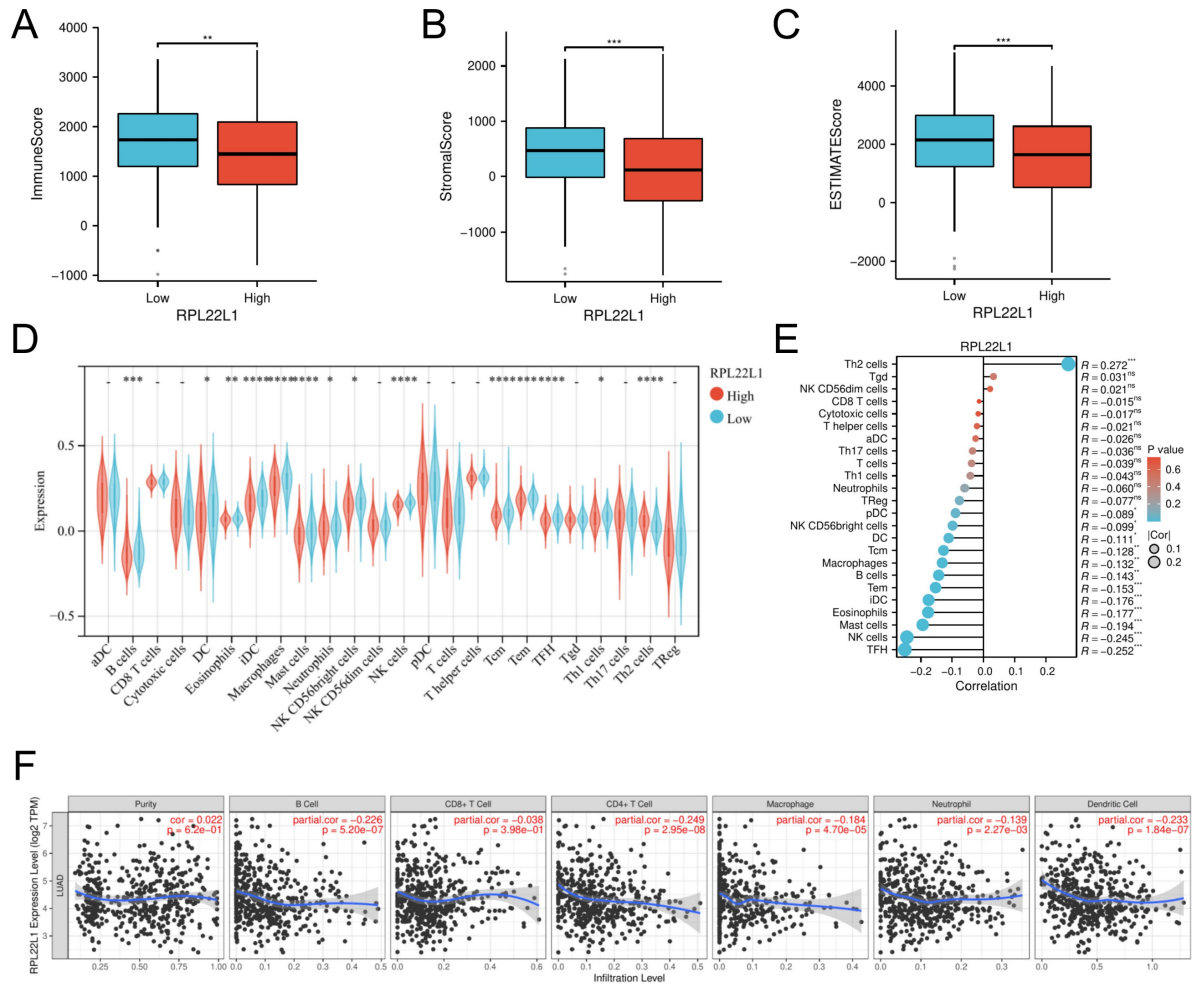
Immune infiltration analysis of RPL22L2 in LUAD. The immune score (A), stromal score (B) and estimate score (C) between the high and low RPL22L1 expression groups. The proportion of 24 immune cell types between low- and high-RPL22L1 group by the single sample Gene Set Enrichment Analysis (ssGSEA) (D-E). Correlation of RPL22L1 mRNA expression with the abundance level of tumor purity, B cell, CD8+ T cell, CD4+ T cell, macrophage, neutrophil and Dendritic cell in the TIMER database (F).

RPL22L1 expression was negatively correlated with the level of infiltration of most immune cells. The ESTIMATE analyses suggested that the high RPL22L1 expression group had lower immune score, stromal score and estimate score than the low RPL22L1 expression group (Figure 6A-C). The ssGESA algorithm was applied to compare the proportion of 24 immune cell types between low and high RPL22L1 groups, and the results showed that RPL22L1 expression was negatively correlated with most immune cells, such as NK cells (r = -0.245, p < 0.001), B cells (r = -0.143, p < 0.01), macrophage (r = -0.132, p < 0.01), iDCs (r = -0.176, p < 0.001), and DCs (r = -0.111, p < 0.05) (Figure 6D-E). The TIMER database similarly confirmed that RPL122L1 was negatively associated with the abundance level of B cells (r = -0.226, p < 0.001), CD8+ T cells (r = -0.038, p < 0.001), CD4+ T cells (r = -0.249, p < 0.001), macrophage (r = -0.184, p < 0.001), neutrophil (r = -0.139, p < 0.001) and dendritic cells (r = -0.233, p < 0.001) (Figure 6F).

### Single-cell level analysis in LUAD TME

**Figure 7:**
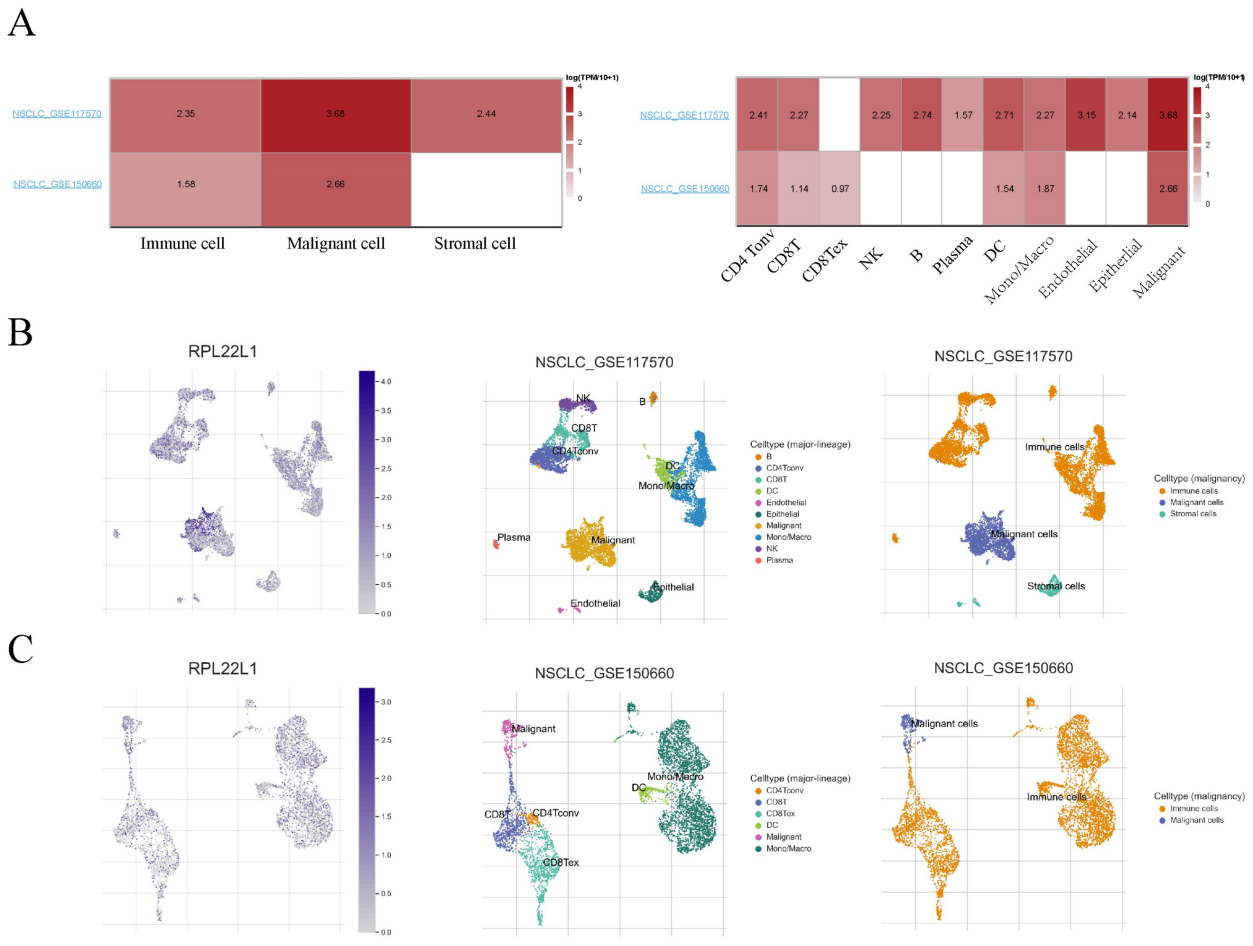
Analysis of RPL22L1 expression in different type of cells at single cell level. Heatmap plot visualizing the average expression of RPL22L1 in various cells (A). Single-cell mapping for visualizing RPL22L1 expression levels in different cell types in the NSCLC_GSE117570 (B) and NSCLC_GSE150660 (C) datasets.

We utilized single-cell datasets (GSE117570 and GSE150660) from the Tumor Immune Single-cell Hub (TISCH) database to analyze the expression of RPL22L1 in LUAD TME. The results showed that RPL22L1 was widely expressed in malignant cells as well as immune cells, such as CD4Tonv cells, CD8+ T cells, CD8Tex cells, B cells, NK cells, DC cells (Figure 7). The expression of RPL22L1 in different types of immune cells indirectly suggests that RPL22L1 might have a potential function in the TME of LUAD.

### Correlation between RPL22L1 expression and drug sensitivity

**Figure 8:**
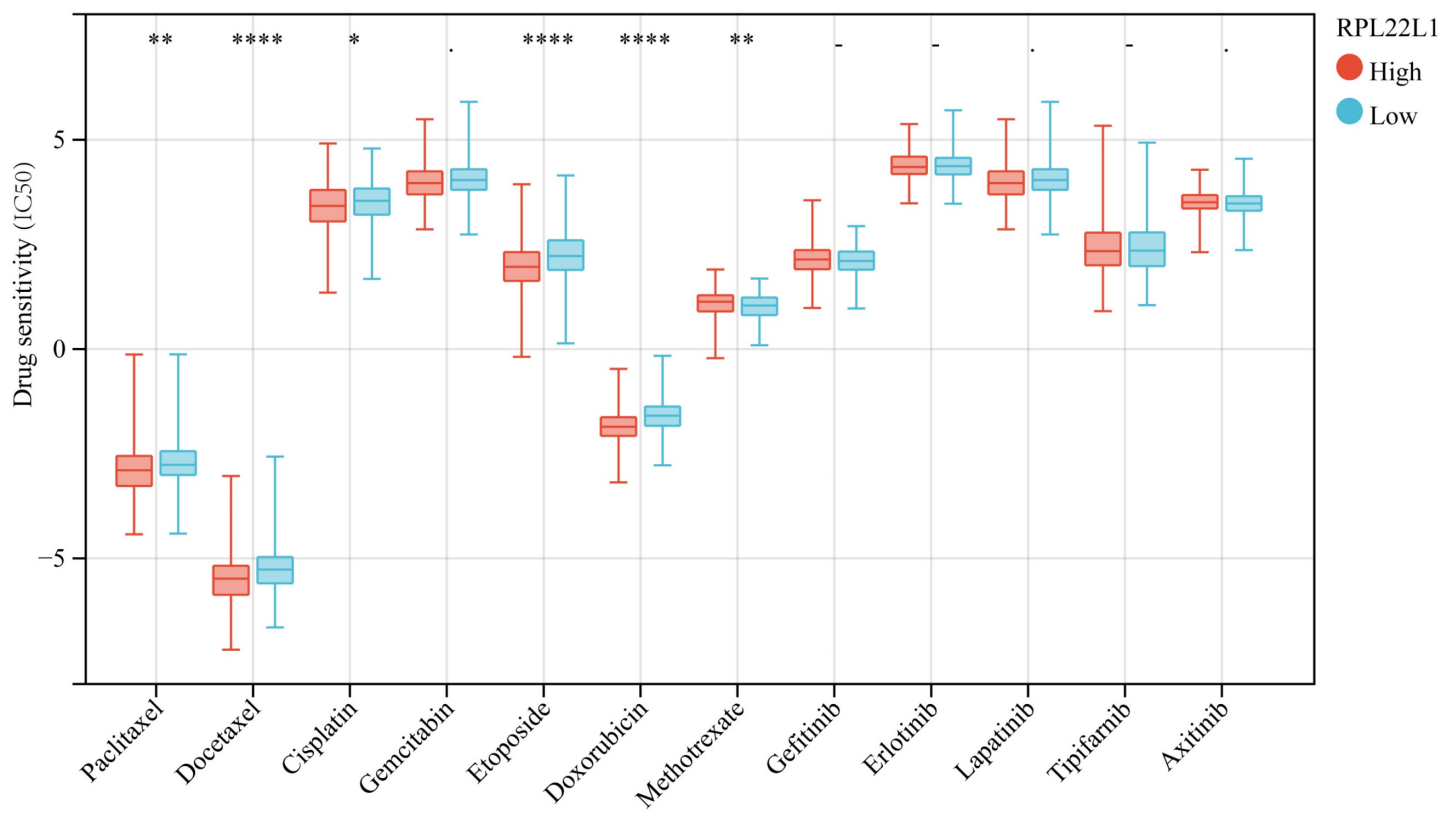
Correlation between RPL22L1 expression and drug sensitivity (IC50) in LUAD.

We analyzed the IC50 of targeted drugs and chemotherapeutic agents according to the expression level of RPL22L1. The results indicated that the level of RPL22L1 was significantly correlated with the sensitivity to multiple drugs. Patients with higher levels of RPL22L1 showed higher sensitivity to paclitaxel, docetaxel, cisplatin, etoposide, and doxorubicin (Figure 8).

## Discussion

A growing number of studies have revealed that ribosomal proteins (RPs) are involved in the regulation of tumorigenesis and development.^5^ There is abnormal expression of RPs in tumors.^34^ RPL22L1 is a member of RPs, and its role in LUAD has not been reported. Therefore, our study is the first preliminary exploration of the significant value of RPL22L1 in LUAD, which provides a basis for further in-depth mechanistic studies.

In the present study, pan-cancer analysis demonstrated that RPL22L1 was significantly up-regulated in most tumors, including LUAD. Survival analysis showed that patients with high RPL22L1 expression had poor prognosis. Multivariate analysis indicated that RPL22L1 was an independent prognostic factor for LUAD. Furthermore, LUAD tissue samples further confirmed that RPL22L1 was highly expressed in LUAD and affected patient prognosis. Besides, we also explored the distribution of mutations associated with RPL22L1 expression. We discovered that Mutations in TP53, KRAS, KEAP1, STK11 and EGFR were significantly associated with RPL22L1 expression. Studies have revealed that these mutated genes are closely linked to the development of tumors.^35^-^37^ Therefore, it is reasonable to assume that RPL22L1 is a potential oncogene that influences the progression of LUAD. However, the functional mechanism of RPL22L1 in LUAD remains unknown.

Several studies have been reported to investigate the mechanism of RPL22L1 in some tumors. A recent study by Chen found that RPL22L1 promoted glioblastoma resistance to temozolomide by activating the STAT3 pathway.^10^ Zhang et al. demonstrated that RPL22L1 promoted resistance to sorafenib in hepatocellular carcinoma through ERK.^11^ A study by Rao indicated that RPL22L1 was associated with poor prognosis and 5-FU resistance in colorectal cancer.^12^ Furthermore, RPL22L1 promoted prostate cancer progression by activating the PI3K/Akt/mTOR signaling pathway.^15^ For ovarian cancer, RPL22L1 could induce epithelial-mesenchymal transition to promote metastasis.^13^ However, there were no studies concerning the specific mechanism of RPL22L1 in LUAD.

To further explore potential regulatory pathways of RPL22L1 in LUAD, we performed GSEA enrichment analysis, which indicted that RPL22L1 high expression was enriched in E2F targets, Myc targets, G2M checkpoint, mTORC1 signaling, DNA repair, Cell cycle, DNA replication, etc. E2F is a complex family of transcriptional regulators whose precise expression and activity are essential for maintaining cell biological behaviors such as the cell cycle. The role of E2Fs in cell proliferation has been widely reported, and their dysfunction contributes to tumor development.^38^ Our study suggested that RPL22L1 was related to the E2F pathway and possibly promoted LUAD progression by mediating the E2F pathway. The Myc pathway and G2M checkpoint were also associated with cell proliferation.^39^, ^40^ In addition, the mTOR signaling is an important signaling pathway in cancer development. It was demonstrated that RPL22L1 promoted prostate cancer progression through mTOR signaling.^41^ Similarly, RPL22L1 has the potential to influence LUAD progression by mediating mTOR signaling. These results implied that RPL22L1 might have a critical role in the progression of LUAD. In addition, we also found that RPL22L1 was associated with some immune-related pathways, including B-cell receptor signaling pathway and humoral immune pathway, suggesting that RPL22L1 may affect the immune microenvironment of LUAD. Thus, RPL22L1 is a promising biomarker for LUAD. The specific functional mechanism of RPL22L1 needs to be further explored in the future.

The tumor microenvironment (TME) is a complex and highly heterogeneous ecosystem. Previous studies have shown that the occurrence and development of cancer are closely related to the TME.^17^, ^42^ Among them, immune cells are an important part of TME, including T cells, B cells, tumor-associated macrophages (TAMs), NK, DCs, tumor-associated neutrophils (TANs), myeloid-derived suppressor cells (MDSCs) and so on. These infiltrating immune cells influence tumor progression and the effectiveness of immunotherapy.^43^ Notably, some ribosomal proteins are involved in regulating immune infiltration in the TME.^20^, ^44^ However, the effect of RPL22L1 in the immune infiltration of LUAD is not fully clear. Our study was the first to reveal that RPL22L1 expression was negatively correlated with the majority of immune cell infiltration, including NK cells, DC cells, B cells, macrophages, CD4+ T cells. Single-cell level analysis revealed that RPL22L1 was expressed not only in malignant tumor cells but also on immune cells. RPL22L1 expression on a wide range of immune cells. On the one hand, it may influence immune cell maturation by regulating their differentiation and development. On the other hand, it may affect the immune microenvironment through negative regulatory pathways. These results indirectly suggest that RPL22L1 may have a potential function in the TME of LUAD. In addition, we also calculated the immune score between the high and low RPL22L1 expression groups utilizing the Estimate algorithm, and as expected, the immune score of the high RPL22L1 expression group was lower compared to those of the low expression group, which further suggested that RPL22L1 might be involved in mediating the suppressive immune microenvironment. In summary, RPL22L1 may promote tumor progression by mediating an immunosuppressive microenvironment and facilitating immune escape.

DNA methylation is an important epigenetic modification in tumorigenesis and development.^45^, ^46^ Our study also found that the methylation level of the RPL22L1 promoter was significantly lower in LUAD compared to normal tissues. In addition, DNA methylation was significantly negatively correlated with RPL22L1 mRNA expression in LUAD. These results suggested that the up-regulation of RPL22L1 expression in LUAD might be due to DNA hypomethylation. Based on methylation profiles, we also discovered that hypomethylation of two CpG sites of RPL22L1 was significantly correlated with poor prognosis. Consequently, methylation of CpG sites may lead to dysregulation of RPL22L1 expression, which in turn affects the prognosis of LUAD patients.

Moreover, we also performed RPL22L1 sensitivity analysis with chemotherapy and targeted drugs. The results indicated that the expression level of RPL22L1 was significantly correlated with sensitivity to multiple drugs. Patients with higher levels of RPL22L1 had higher sensitivity to paclitaxel, docetaxel, cisplatin, etoposide, and doxorubicin. It is of great significance for clinical therapeutic decision making.

Finally, although our study preliminarily revealed a link between RPL22L1 and LUAD, some limitations were observed. Firstly, lack of in vivo and in vitro functional experiments to validate the potential mechanism of RPL22L1 in LUAD. Secondly, We utilized data from public databases and lacked detailed clinical information, which makes it impossible to determine whether the enrolled patients may also suffer from other diseases that may significantly affect the RPL22L1 expression status. Finally, although our study showed that RPL22L1 expression was closely associated with immune cell infiltration in LUAD, direct evidence of RPL22L1 involvement in cell immune infiltration was lacking. These issues deserve further exploration in the future.

## Conclusion

In conclusion, our study confirmed that RPL22L1 was overexpressed in LUAD, and its expression was significantly correlated with clinical features, prognosis, DNA methylation, TME, and drug sensitivity. RPL22L1 is a promising biomarker for the diagnosis, treatment, and prognosis of LUAD. However, the mechanism of RPL22L1 in LUAD needs to be further explored and validated in the future.

## Figures and Tables

**Table 1:** Characteristic between low and high expression of RPL22L1 in LUAD from the TCGA database.

Characteristics	High expression	Low expression	P value
n	270	269	
Age, n (%)			0.956
≤65	130 (48.1%)	127 (47.2%)	
>65	131 (48.5%)	132 (49.1%)	
Unknown	9 (3.3%)	10 (3.7%)	
Gender, n (%)			0.001
Female	126 (46.7%)	163 (60.6%)	
Male	144 (53.3%)	106 (39.4%)	
T stage, n (%)			0.072
T1/T2	230 (85.2%)	238 (88.5%)	
T3/T4	40 (14.8%)	28 (10.4%)	
Unknown	0 (0%)	3 (1.1%)	
N stage, n (%)			< 0.001
N0	174 (64.4%)	176 (65.4%)	
N1/N2/N3	95 (35.2%)	78 (29%)	
Unknown	1 (0.4%)	15 (5.6%)	
M stage, n (%)			0.006
M0	193 (71.5%)	172 (63.9%)	
M1	17 (6.3%)	8 (3%)	
Unknown	60 (22.2%)	89 (33.1%)	
Pathologic stage, n (%)			0.039
Stage I/II	199 (73.7%)	222 (82.5%)	
Stage III/IV	67 (24.8%)	43 (16%)	
Unknown	4 (1.5%)	4 (1.5%)	

**Table 2:** Univariate and multivariate analysis of OS for LUAD in the TCGA database.

Characteristics	Univariate analysis			Multivariate analysis	
	Hazard ratio (95% CI)	P value		Hazard ratio (95% CI)	P value
Age					
≤65	Reference				
>65	1.217 (0.911 - 1.625)	0.185			
Unknown	0.344 (0.108 - 1.094)	0.071			
Gender					
Female	Reference				
Male	1.088 (0.817 - 1.449)	0.565			
T stage					
T1/T2	Reference			Reference	
T3/T4	2.354 (1.616 - 3.429)	< 0.001		1.643 (1.074 - 2.515)	**0.022**
Unknown	3.748 (0.921 - 15.247)	0.065		5.007 (0.661 - 37.933)	0.119
N stage					
N0	Reference			Reference	
N1/N2/N3	2.547 (1.904 - 3.407)	< 0.001		2.141 (1.514 - 3.028)	**< 0.001**
Unknown	1.190 (0.436 - 3.247)	0.734		0.839 (0.206 - 3.425)	0.807
M stage					
M0	Reference			Reference	
M1	2.146 (1.256 - 3.668)	0.005		1.188 (0.640 - 2.205)	0.585
Unknown	0.833 (0.582 - 1.191)	0.317		0.992 (0.686 - 1.433)	0.965
Pathologic stage					
Stage I/II	Reference			Reference	
Stage III/IV	2.703 (1.988 - 3.675)	< 0.001		1.394 (0.898 - 2.163)	0.139
Unknown	0.712 (0.176 - 2.886)	0.635		0.540 (0.133 - 2.199)	0.390
RPL22L1					
Low expression	Reference			Reference	
High expression	1.522 (1.133 - 2.046)	0.005		1.507 (1.104 - 2.058)	**0.010**

**Table 3:** Univariate and multivariate analysis of OS for LUAD in our cohort.

Characteristics	Univariate analysis			Multivariate analysis	
	Hazard ratio (95% CI)	P value		Hazard ratio (95% CI)	P value
Age					
≤65	Reference				
>65	1.614 (0.938 - 2.779)	0.084			
Gender					
Female	Reference				
Male	1.169 (0.691 - 1.978)	0.560			
T stage					
T1-2	Reference			Reference	
T3-4	3.245 (1.502 - 7.011)	0.003		1.894 (0.729 - 4.925)	0.190
N stage					
N0	Reference				
N1/N2/N3	1.538 (0.897 - 2.637)	0.118			
Pathologic stage					
Stage I/II	Reference			Reference	
Stage III/IV	2.292 (1.242 - 4.229)	0.008		1.857 (0.862 - 3.999)	0.114
RPL22L1					
Low expression	Reference			Reference	
High expression	1.895 (1.112 - 3.231)	0.019		1.908 (1.115 - 3.266)	**0.018**
